# Supplementary material for: Meta-Transcriptomic Discovery of a Divergent Circovirus and a Chaphamaparvovirus in Captive Reptiles with Proliferative Respiratory Syndrome
Source: Viruses. 2020 Sep 25;12(10):1073. doi: 10.3390/v12101073 (PMC7600432; doi:10.3390/v12101073)
Supplement: Supplementary file 1 [file viruses-12-01073-s001.pdf]

SI Table1. Presentation, pathology in central bearded dragon morbidity and mortality event, 2014.

Case ID	Signalment	Gross lesions	Proliferative respiratory Epithelium	Pneumonia I/N	Eosinophilic Cytoplasmic IB
10043.1	Male/Adult	Miliary white hepatic foci. Pleural petechiae, pulmonary congestion, mucoid yellow exudate in mesobronchus. Small testes. Fair body condition.	4D	4D/2F	Lung, kidney, liver
10043.2	Female/Adult	Mottled hepatic parenchyma. Pulmonary congestion. Multifocal haemorrhagic ova. Fair body condition.	1D	2MF/2MF	Liver
10043.3	Female/Adult	Mulitofocal haemorrhagic ova. Good body condition	1D	3D/0	0
10043.4	Male/Adult	Dehydrated. Hepatic atrophy. Blood surrounding bolus of food in large intestine. Small testes. Poor body condition.	4D Binucleate cells and syncytia	1D/1D	Lung, kidney, liver
10043.5	Female/Adult	Mottled hepatic parenchyma. Pulmonary congestion and unilateral mesobronchial blood clot. Small ova. Poor body condition.	3D	3D/3D	Lung, nerve cell body

Proliferation of respiratory epithelium graded on a scale of 0-4 in ascending severity. Inflammation (I) graded on a scale of 0-4 in ascending severity. Necrosis (N) graded on a scale of 0-4 in ascending severity. D – diffuse, S – segmental, MF – multifocal, F – focal tracts. IB: inclusion bodies. Pulmonary and hepatic inflammation were graded on a scale of 0-4, where 0 indicated no discernible lesions, 1 represented scattered leukocytes and 4 represented severe and extensive heterophilic inflammation. Pulmonary and hepatic necrosis were graded on a scale of 0-4 where 0 indicated no discernible necrosis, 1 represented mild single cell degeneration or necrosis, and 4 characterized extensive caseating necrosis.
